# Design and Development of a Traveling Wave Ferro-Microfluidic Device and System Rig for Potential Magnetophoretic Cell Separation and Sorting in a Water-Based Ferrofluid

**DOI:** 10.3390/mi14040889

**Published:** 2023-04-21

**Authors:** Rodward L. Hewlin, Maegan Edwards, Christopher Schultz

**Affiliations:** 1 Center for Biomedical Engineering and Science (CBES), Department of Engineering Technology and Construction Management (ETCM), University of North Carolina at Charlotte, Charlotte, NC 28223, USA; 2 Applied Energy and Electromechanical Systems (AEES), Department of Engineering Technology and Construction Management (ETCM), University of North Carolina at Charlotte, Charlotte, NC 28223, USA

**Keywords:** cellular, circuit board, design, diagnosis, diseases, ferro-microfluidic, ferrofluid, frequency, heat, magnetic, manipulation, medical, non-magnetic, separation, traveling wave

## Abstract

The timely detection and diagnosis of diseases and accurate monitoring of specific genetic conditions require rapid and accurate separation, sorting, and direction of target cell types toward a sensor device surface. In that regard, cellular manipulation, separation, and sorting are progressively finding application potential within various bioassay applications such as medical disease diagnosis, pathogen detection, and medical testing. The aim of this paper is to present the design and development of a simple traveling wave ferro-microfluidic device and system rig purposed for the potential manipulation and magnetophoretic separation of cells in water-based ferrofluids. This paper details in full: (1) a method for tailoring cobalt ferrite nanoparticles for specific diameter size ranges (10–20 nm), (2) the development of a ferro-microfluidic device for potentially separating cells and magnetic nanoparticles, (3) the development of a water-based ferrofluid with magnetic nanoparticles and non-magnetic microparticles, and (4) the design and development of a system rig for producing the electric field within the ferro-microfluidic channel device for magnetizing and manipulating nonmagnetic particles in the ferro-microfluidic channel. The results reported in this work demonstrate a proof of concept for magnetophoretic manipulation and separation of magnetic and non-magnetic particles in a simple ferro-microfluidic device. This work is a design and proof-of-concept study. The design reported in this model is an improvement over existing magnetic excitation microfluidic system designs in that heat is efficiently removed from the circuit board to allow a range of input currents and frequencies to manipulate non-magnetic particles. Although this work did not analyze the separation of cells from magnetic particles, the results demonstrate that non-magnetic (*surrogates for cellular materials*) and magnetic entities can be separated and, in some cases, continuously pushed through the channel based on amperage, size, frequency, and electrode spacing. The results reported in this work establish that the developed ferro-microfluidic device may potentially be used as an effective platform for microparticle and cellular manipulation and sorting.

## 1. Introduction

Lab-on-a-chip (*LOC*) devices for cellular manipulation and sorting are now being used in a variety of research applications involving cancer diagnosis, pathogen detection, and rapid genomic testing [1,2]. Dielectrophoresis (*DEP* ), immunolabeling, magnetic bead separation, and laminar flow-based separation are some of the common techniques utilized [3,4,5,6,7]. In addition to current methods for cellular manipulation at the nanoscale, separation methods based on magnetic beads require labeling, as well as multiple and prolonged incubation and wash cycles. Conversely, schemes based on electric fields suffer from high voltage requirements, have a performance that is highly dependent on the ionic strength of the solution [8,9], and negatively affect cell metabolism and vitality by polarizing their membranes [10].

Using functionalized magnetic beads to separate target molecules and cells can overcome these challenges using magnetic fields instead of electric fields. There have been various works reported on using magnetic fields to separate cells in continuous microfluidic flow. Sutermaster and Darling investigated the ability of separating defined mixtures of alkaline phosphatase liver/bone/kidney (*ALPL*)-expressing and non-expressing cell types using magnetic fields [11]. The results from this work showed that initial magnetic-activated cell sorting (*MACS*) runs performed using the manufacturer’s recommended antibody and microbead concentrations produced inaccurate ALPL+ vs. ALPL− cell splits compared to fluorescence-activated cell sorting (*FACS*) when ALPL+ cells were present in larger proportions (>~25%). Shamloo et al. designed and developed two centrifugal microfluidic devices for the isolation of rare cancer cells [12]. These devices were tested at different disk rotational speeds, and it was reported that the passive design could isolate MCF-7 cells with a recovery rate of 76%.

Yang et al. investigated separating circulating tumor cells using a ferrohydrodynamic approach [13]. Liu et al. reported a separation method termed quantitative ferrohydrodynamic cell separation (*qFCS*). In this work, the authors were able to achieve multimodal rare cell sorting and simultaneous antigen profiling at a ∼30,000 cells/min throughput with a 96.49% recovery rate and a 98.72% purity of recovered cells [14]. Although there has been some success in using this technique for separating living cells, the reported downsides of this technique are the lengthy incubation times and wash cycles and the difficulty of removing the label post priori [15,16,17]. The deterministic hydrodynamics approach, as reported in the work of Davis et al. [18], can achieve high-resolution separation without the use of any electromagnetic fields. However, high throughput with this device requires high-resolution lithography on a large area, keeping the cost per device high. There is also a small number of works that have investigated separating cells and magnetic materials in microfluidic devices using a traveling wave magnetic field [19,20,21]. More research is needed to investigate the ability to separate cells in continuous microfluidic flow using a traveling wave magnetic field [22].

To address performance limitations and the current knowledge gaps, our group aims to design and develop a microfluidic platform based on ferro-hydrodynamics for the manipulation and separation of cells and microorganisms within dynamic ferrofluids under an applied transient field. This work is similar to the work of Kose et al. [19]. This technique involves using a water-based ferrofluid as a uniform magnetic environment that surrounds the cells within a microfluidic channel. Cells and other nonmagnetic particles within the ferrofluid act as “magnetic voids” [23], in a manner comparable to electronic holes in a semiconductor. An externally applied magnetic field gradient attracts magnetic nanoparticles and causes nonmagnetic nanoparticles or cells to be effectively pushed away [24,25,26,27]. This principle has been applied in the author’s earlier works to capture non-magnetic microparticles between magnetic film islands and in other microfluidic devices [28,29]. 

The design reported in this work consists of integrated copper electrodes that carry alternating currents in quadrature to generate programmable magnetic field gradients locally for magnetic manipulation and separation of magnetic nanoparticles and non-magnetic microparticles within the ferrofluid. This paper details the design, methodology, and results. The key contributions of this work are:

1. A method for tailoring preferred size range (10–20 nm) magnetic cobalt ferrite magnetic nanoparticles.
2. The development of a ferro-microfluidic device for potentially separating cells and magnetic microparticles without detrimental thermal effects.
3. The development of a water-based ferrofluid with magnetic and non-magnetic particles as surrogates for biological cells.
4. The design and development of a rig for producing the electric field within the ferro-micro fluidic device for magnetizing the magnetic nanoparticles and manipulating nanoparticles in static and dynamic flow while efficiently removing heat from the electrode base.
5. A method for measuring the surrounding fluid and particle velocity, vorticity, and characterizing particle dynamics in the developed ferrofluid.

The next section discusses the materials and methods of this work.

## 2. Materials and Methods

This section of the paper provides an overview of the materials and methodology of this work. The order of this section is as follows (1) the ferro-microfluidic device and system rig design and development and assembly methodology, (2) the heat dissipation system design methodology, and (3) the ferrofluid tailoring and preparation methodology. The next section introduces the device development and assembly methodology. 

### 2.1. Device Development and Assembly Methodology

**Figure 1:**
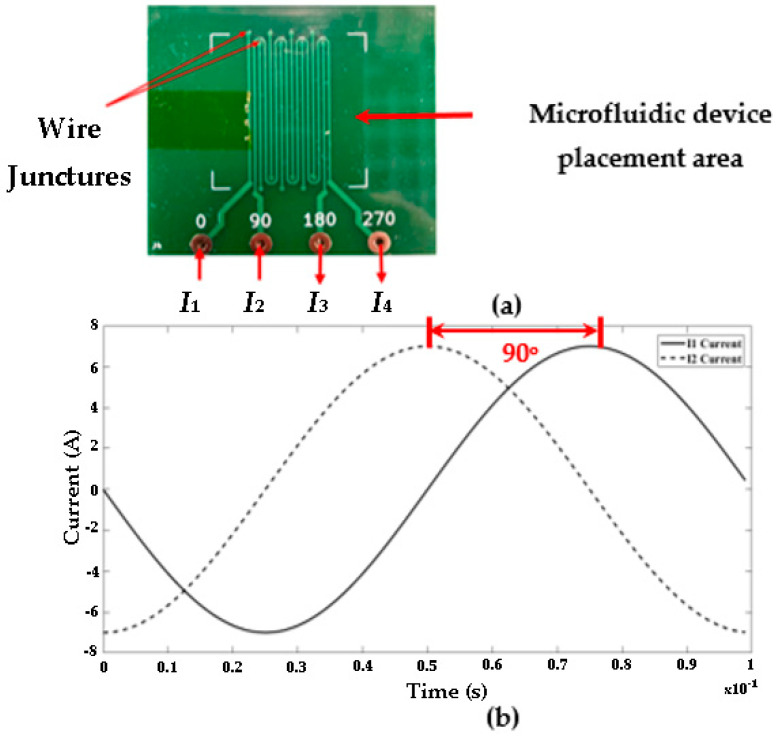
Overview of the programmable chip platform and current waveform: (**a**) Photo of the first fabricated programmable electronic chip base. The arrows going in represent the current amplitudes supplied to the chip at a 90° phase, and the arrows coming from the chip represent the output currents at a 90° phase difference. (**b**) The current sinusoidal input current waveforms for *I*_1_ and *I*_2_.

The ferro-microfluidic particle manipulation device used in our experiments consists of two parts: the microfluidic channel and the underlying programmable copper electrode circuit board chip. The electrodes (30 µm high, 300 µm wide, and 2 cm in length) were fabricated by an external consultant (*Sigenics Inc., Chicago, IL, USA*). The electrodes were fabricated via wet etching the copper layer of a thermal-clad printed circuit board (*on an insulated metal substrate*) through a photoresist mask. The electrodes were bonded via internal board wire junctions to complete the quadrature electrode circuit. A photo of the developed chip is shown below in Figure 1.

In this work, a traveling wave magnetic field is generated in the channel by applying alternating currents in quadrature to a single layer of electrodes. A plot of the input current profiles for both inputs at 7 A and 10 kHz is shown in Figure 1b. The microfluidic channel (20 µm to 100 µm high, 1 mm to 3 mm wide, and 2 cm to 3 cm long) was prepared from polydimethylsiloxane (*PDMS*) stamps through soft lithography and was bonded to a glass slide that acts as an insulating layer between the channel and the electrodes. The channel height was chosen to be well below the optimum for localized ferrohydrodynamic flow to minimize its potential effects on microparticle migration. For PDMS removal, the PDMS is allowed to cure, and the microfluidic device is easily peeled off from the salinized SU-8 master. The fluidic connection holes are punched with a syringe needle. The SU-8 master can be reused as many times as needed to create additional PDMS devices. 

The PDMS channel device was treated via plasma treatment (*in a plasma cleaner chamber from Harrick Scientific, Pleasantville, NY, USA*) at 90 mmHg O_2_ partial pressure with 18 W power for one minute. This enables the PDMS to be rendered hydrophilic and allows the PDMS and glass components to create a strong bond. The PDMS was then bonded to the glass slide. This attachment is sustained via oxygen bonds between silanol groups, formed on the PDMS surface via plasma treatment. 

**Figure 2:**
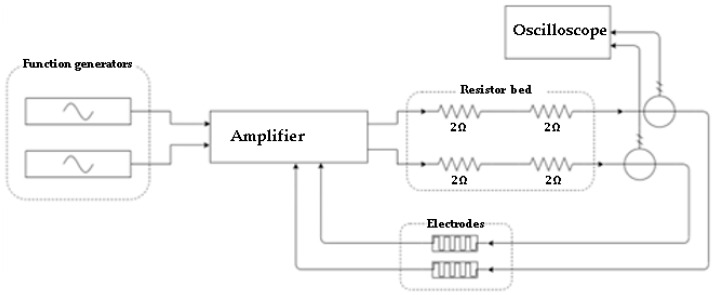
Schematic diagram of the experimental rig electronic components.

Once the microfluidic device is fully assembled, it is placed over a cooling water block (*the cooling block is discussed in Section 2.2*) with a thin layer of a silver-thermal conductive paste and glue (*8349TFM MG Chemicals Thermal Adhesive*) to dissipate heat. The electrode pads are connected to a stereo-amplifier (*Crown Com-Tech 410, 2-Channel Power Amplifier, Los Angeles, CA, USA*) with connection cables to both channels. A resistor bed was created from 2 Ω resistors wired in series to avoid large current pulses during the startup of the stereo-amplifier. A schematic of the electronics setup is shown below in Figure 2.

**Figure 3:**
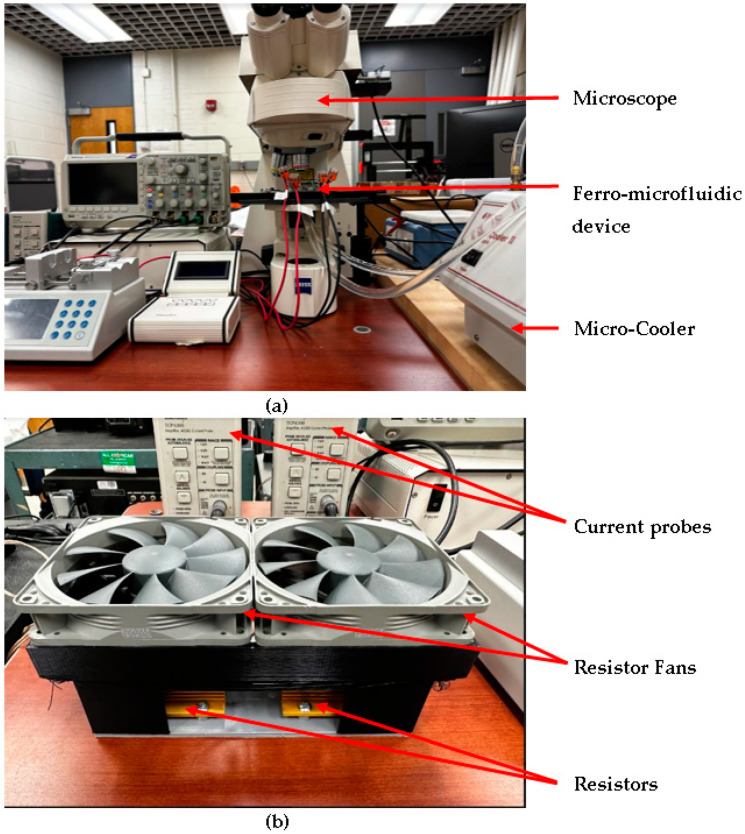
Photograph of the experimental rig setup: (**a**) microscope and cooling system setup with the oscilloscope and (**b**) the 3D-printed resistor housing and cooling setup.

A 3D resistor housing was 3D printed to cover the resistors, and two CPU fans were installed on top of the housing to cool the resistors during experimental runs. Two function generators from Agilent (*33220A Function/Arbitrary Waveform Generator, Santa Clara, CA, USA*) are phase-locked to each other to generate the sinusoidal signal, and the resulting current is amplified with the stereo-amplifier. The current is measured by two current probes from Tektronix (*TCPA 300, TCP 305, Richardson, TX, USA*) connected to the cables. An oscilloscope (*Tektronix, TDS 2014, Digital Oscilloscope, Beaverton, OR, USA*) is connected to the current probes and monitors the current and frequency. A Zeiss Axioplan 2 microscope outfitted with a camera is used to record images of the ferrofluid and fluorescent microparticle mixture dynamics under an applied field. The current amplitude and frequency as well as the phase difference between the two channels are controlled by a MATLAB program. A photo of the experimental setup is shown in Figure 3. The next section discusses the heat dissipation system design methodology.

### 2.2. Heat Dissipation System Design, Development and Evaluation Methodology

The programmable electronic chip base is designed to operate with current levels of 1–7 A and cover a large frequency spectrum range (10 Hz–1 kHz). At these current levels, heating of the device and critical thermal change to the electrodes are a vital concern. We have conducted a simple initial computational thermal heat load analysis using Solidworks^TM^ Thermal Simulation package v.22 to provide a baseline perspective of the temperature range for the low and high heat loads. In this analysis, we conducted a low and high thermal power load assessment based on the minimum and maximum current settings of 1 A and 7 A. 

**Figure 4:**
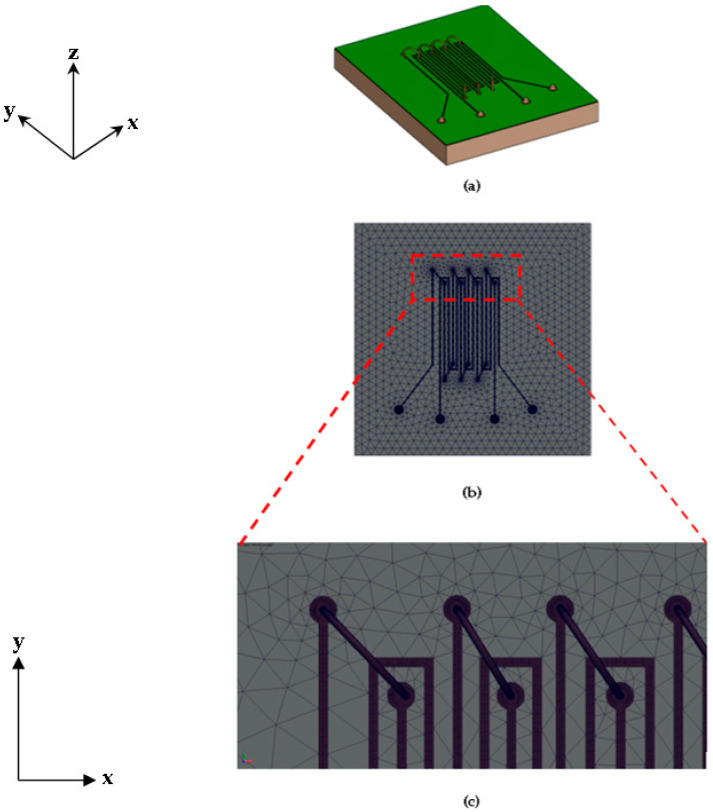
Computational model of the electronic chip and copper base for the heat dissipation analysis: (**a**) CAD model, (**b**) meshed model, and (**c**) close view of electrode meshing.

For pre-processing, we deployed the CAD model into the thermal simulation package of Solidworks^TM^ and applied PCB PR4 thermal material properties to the circuit board and copper thermal material properties to the electrodes. An ambient initial temperature setting of 300 K was applied to the top and side faces of the model. A convection coefficient (*also called the film coefficient*) of 25 (W/m^2^)/K was applied. The value of the convection coefficient corresponds to natural convection taking place without a fan. Two heat load settings were applied to the electrodes for a low setting of 2 W and a high setting of 8 W. We assumed that the electrodes would experience a 0.5 Ω resistance. Using the formula for electrical power, these settings are representative of 2 and 4 A. For meshing, a mesh control of 3 µm was applied to the electrodes, and the final mesh was set to the finest meshing scheme. The resulting mesh contained 1,171,391 tetrahedral elements. This was the maximum number of elements that could be used to avoid memory and RAM issues. Figure 4 below shows the model and meshed result.

**Figure 5:**
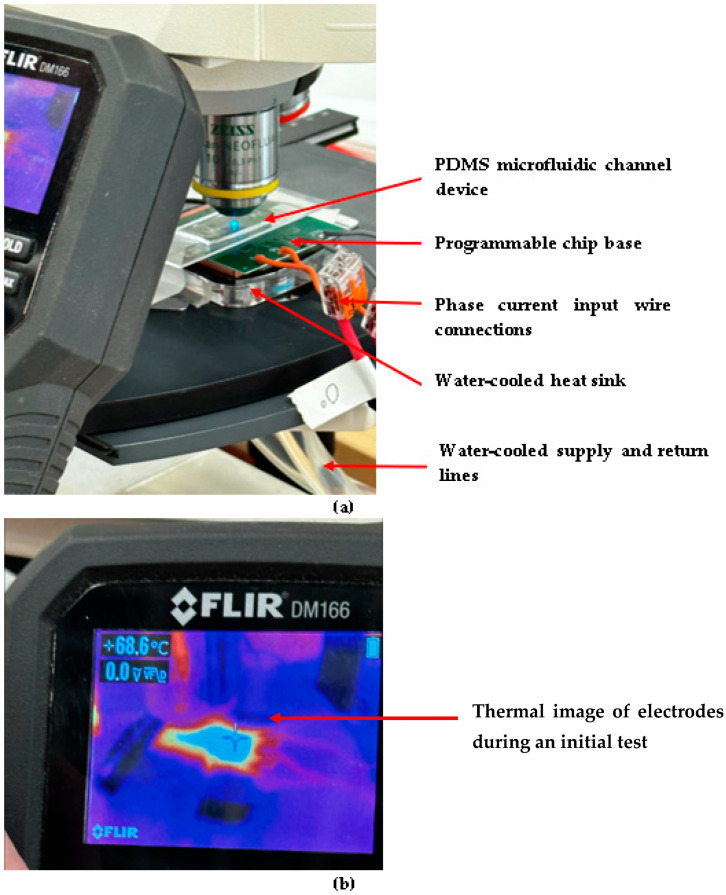
System rig and water-cooled heat dissipation setup: (**a**) full system with the ferro-microfluidic device and water block and (**b**) the thermal image of the electrodes with an input current.

**Figure 6:**
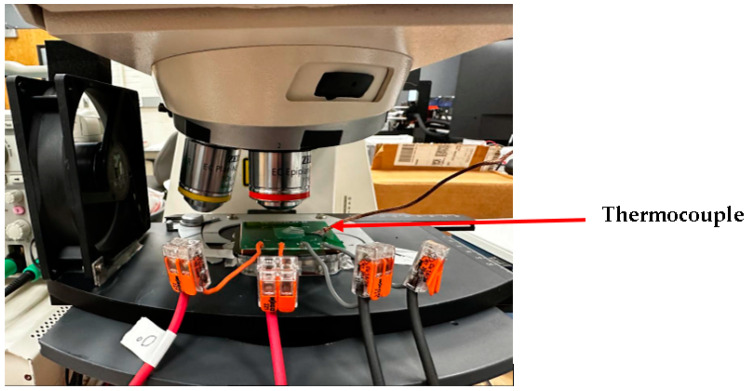
Thermal experiment setup.

The Results section discusses the results of the simulation. We have also evaluated two designs developed to enhance heat dissipation and prevent burning of the electrodes. In this regard, the wires that were used to connect each electrode pad are connected via soldered connections and are covered with a high thermal conductance epoxy. For our initial design, we constructed a forced convection setup consisting of a 3D-printed stand outfitted with a copper heat sink and central processing unit (*CPU*) fan. A series of tests were run to determine the temperature response of the electrodes to the applied current. A National Instruments cDAQ 9133 data acquisition system and thermocouple were used to obtain temperature profile data for current settings. The thermocouple was tightly connected directly atop the electrodes to measure the temperature change. The results section provides the results from this test, and this setup did prove to be ineffective. We later incorporated a cooling water system into the experimental setup rig. The cooling system consisted of a Bewinner 800 L/H mini water-cooling pump and clear flexible lines to circulate water, an Aveks CPU 50 mm water block to cool the chip, and a Boekel Benchtop Micro-cooler II to cool the circulating water. For later thermal experiments, a Neslab Endocal RTE-5B Heated/Refrigerated Circulating Bath was used to cool the cooling block with water as the working fluid. Figure 5 below shows a photograph of the rig set up with the water-cooled pump and CPU water block. A CPU fan was also added in the final setup shown in Figure 6. A FLIR DM166 imaging multimeter was used to monitor the infrared temperature profiles, and a thermocouple was used similar to the initial design to evaluate the temperature profiles. 

For the water-cooled heat dissipation design system setup experiments, the mini cooler was set to a temperature setpoint of −20 °C in which water circulated throughout flexible lines and the water block during the experiments. The actual cooling temperature was taken to be 0 °C. The temperature was recorded periodically for 5 min while currents of 0.5 to 6 A were applied to the electrodes using an excitation frequency of 1 kHz. The results section discusses the results of the experiments. 

The next section discusses the ferrofluid tailoring methodology and the channel device preparation methodology. 

### 2.3. Ferrofluid Tailoring and Ferro-Microfluidic Device Preparation

Tailoring the ferrofluid for this work consisted of two parts: (1) Developing the magnetic cobalt ferrite microparticles, and (2) suspending the cobalt ferrite microparticles and fluorescent microparticles in deionized water for the resultant aqueous ferrofluid. Section 2.3.1 describes the cobalt ferrite microparticle fabrication process. We ran two batch processes to determine a recipe for small (10–20 nm diameter) size particles. 

#### 2.3.1. Cobalt Ferrite Microparticle Tailoring Process

Ferric chloride, cobalt chloride (98 +% purity), and sodium hydroxide were used to develop the magnetic cobalt ferrite particles. Eleven grams of oleic acid of HPCL grade was used as a surfactant. Double-distilled, de-ionized water was used as a solvent. All the materials were of reagent grade and used without further purification. The fabrication process began with mixing a 25 mL (0.4 M) solution of iron chloride and a 25 mL (0.25 M) solution of cobalt chloride in double-distilled, de-ionized water. The deionized distilled water was used as a solvent to avoid the production of impurities in the final product. A 3 M (25 mL) solution of sodium hydroxide was prepared and slowly added to the salt solution dropwise. The pH of the solution was constantly monitored as the NaOH solution was added. The reactants were constantly stirred using a magnetic stirrer until a pH level of 11–12 was reached. 

The liquid precipitate was then brought to a reaction temperature of 80 °C and stirred for one hour. The product was then cooled to room temperature. To obtain free particles from sodium and chlorine compounds, the precipitate was then washed twice with distilled water and then with ethanol to remove the excess surfactant from the solution. To isolate the supernatant liquid, the beaker contents were then centrifuged for 15 min at a given rpm (*later discussed in the results*) using a centrifuge. The supernatant liquid was then decanted and centrifuged until only a thick black precipitate remained. The precipitate was then dried overnight at 450 °C in an oven to remove excess liquid. The acquired substance was then ground into a fine powder. At this stage, the product (*CoFe_2_O_4_*) contains some associated water (up to 10 wt%), which was then removed by heating at 600 °C for ten hours. The final product obtained was then confirmed by X-ray diffraction to be magnetic nanoparticles of cobalt ferrite (*CoFe_2_O_4_*) with an inverse spinel structure. Section 3 displays the X-ray diffraction and SEM images of the fabricated cobalt ferrite microparticles.

The tailoring of the ferrofluid involved suspending the cobalt ferrite microparticles in deionized water. The resulting ferrofluid had a viscosity of 1.75 cP at 20 °C. Fluorescent microparticles ranging in diameter from 1–10 µm, depending on the experiments run, were added. Before introducing the ferrofluid/microsphere mixture into the microfluidic device, the channel was washed with a 1% triton-X solution in water for 10 min to minimize microparticle attachment to the PDMS microchannel walls. During experimental runs, the fluorescent particles were tracked and characterized with particle image velocimetry (*PIV*) and the open-source code PIVLAB, as used in our previous works [30,31,32,33,34]. The next section presents the conclusions of this work.

## 3. Results

This section of the paper provides a discussion of the results obtained from the heat dissipation computational analysis, the results from the cobalt ferrite nanoparticle and ferrofluid tailoring procedure, and the initial particle dynamics analysis for the ferro-microfluidic device characterization. We begin with the results from the heat dissipation computational analysis. 

### 3.1. Heat Dissipation System Design Results

In this work, we conducted a simple initial computational thermal heat load analysis using Solidworks^TM^ Thermal Simulation package v.22 to provide a baseline perspective of the temperature range for the low and high heat loads. For heat conduction computational analyses, the Solidworks Thermal Simulation package solves the Fourier heat conduction equation below in Equation (1) in a finite element form:

(1) $$\rho c_{p}\frac{\partial T}{\partial t}=k\left(\frac{\partial^{2}T}{\partial x^{2}}+\frac{\partial^{2}T}{\partial y^{2}}+\frac{\partial^{2}T}{\partial z^{2}}\right)$$

where *ρ* is the material density, *c_p_* is the material specific heat, *k* is the thermal conductivity, and *x*, *y*, and *z* are the three-dimensional spatial directions of heat transfer.

**Figure 7:**
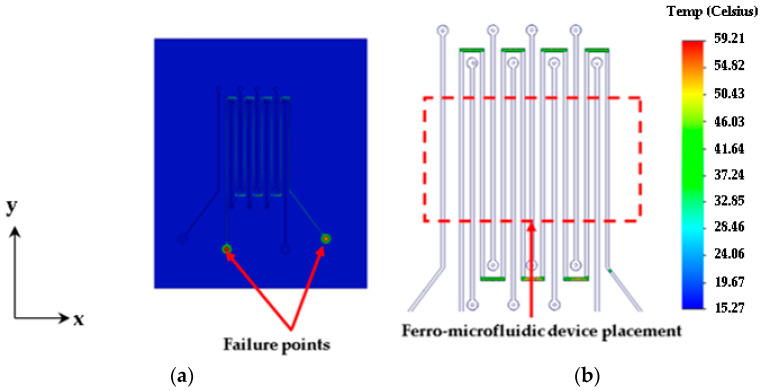
Thermal simulations at 2 W for 3 s: (**a**) thermal contours and (**b**) thermal iso-contours. The dashed lines in the iso-contour represent the area where the ferro-microfluidic device will sit. The black arrow on the color scale represents the temperature threshold for cell damage.

For this analysis, we conducted a low and high thermal power load assessment based on the minimum and maximum current settings of 1 A and 7 A. Both the low and high load simulations were transient and were run for a total time of 3 s with 0.01 s timesteps. The simulation took a total time of 1 h to run with the default thermal finite element (*FEM*) and transient solver. Figure 7 below shows a temperature and thermal iso-contour for the low load simulation for a total time of 3 s.

As shown in Figure 7, the temperature distribution in the electrodes is uneven from the input pads throughout the two electrode profiles (*bonded electrodes*) to the output pads. An interesting phenomenon observed is that the highest load point occurs at the wiring input and output pads. Several simulations were conducted with varying mesh sizes, and we noticed the same trends in heating regardless of the mesh size and type. This phenomenon may be due to the fact that a heat-conductive object with a wider surface area has more surface area to act as a conduit for conducting and transferring heat [35,36]. In this case, the rate of heat transfer is directly proportional to the surface area through which the heat is being conducted. Additionally, when comparing the pads to the actual electrodes, the pads will indeed conduct more heat than the electrodes during current inputs. 

**Figure 8:**
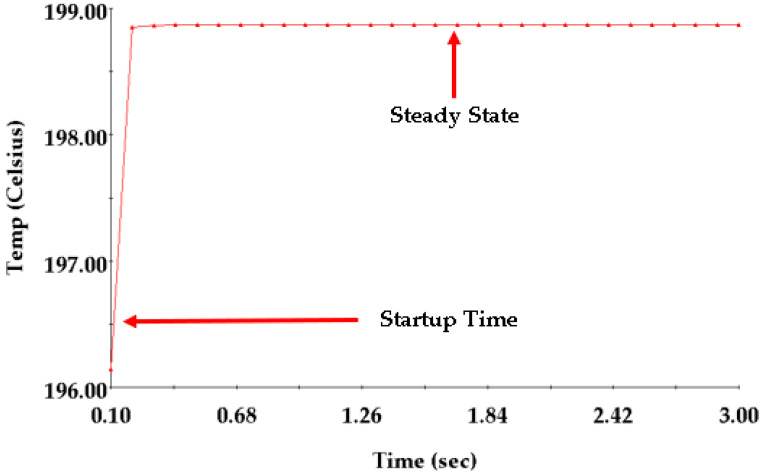
Temperature vs. time probe profile plot at the first electrode for 7 A without a heat sink and proper cooling.

For the low heat load analysis, the highest temperature observed at 3 s was 59.21 °C. The temperature is well above the temperature of cell damage. This temperature remains constant for the duration of the current input, indicating that the simulation reaches a steady state at 3 s. The thermal iso-contour shown in Figure 7b also shows that the highest temperature in the electrodes is observed in the areas where the electrodes make sharp turns. This phenomenon is due to conductivity resistance. Sharp corners influence heat transfer similar to sharp bends in fluid pipes, which create energy losses and flow disturbances. We took this into consideration for the development of the chip base. For the high heat load case (7 A), we noticed that the temperature climbs instantly at 0.1 s and reaches a steady state at 0.2 s. Figure 8 below shows a temperature probe profile plot at a nodal point in the first electrode. From Figure 8, it is observed that without a heat sink and proper cooling, the electrodes will degrade fast under high heat loads.

**Figure 9:**
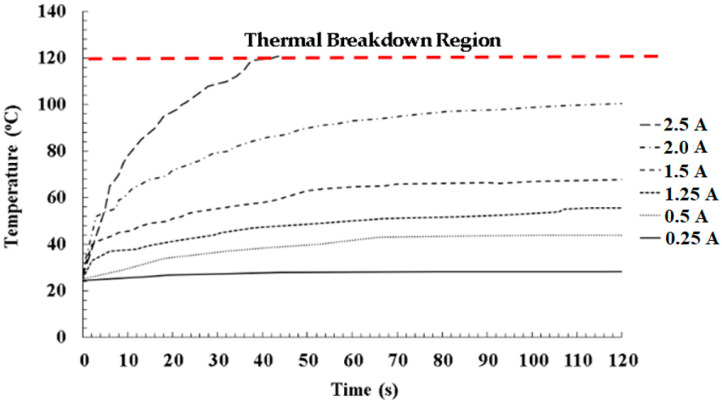
Temperature vs. time profile of the electrodes at various current settings. The dashed line represents the thermal breakdown region for the copper electrodes.

After running the computational simulations, we conducted four forms of experimental thermal analysis studies. As mentioned previously, the electronic chip is designed and expected to operate with current levels of 1–7 A and cover a large frequency spectrum (10 Hz–100 kHz). We first conducted a thermal heating analysis, where we experimentally measured the temperature of the electronic chip base as a function of time, increasing amperage, and at a maximum frequency of 1 kHz. This analysis consisted of powering the electrodes and recording the temperature of the electrodes without a heat sink or cooling device. Figure 9 shows a plot of the temperature profile at each current setting at 1 kHz.

**Figure 10:**
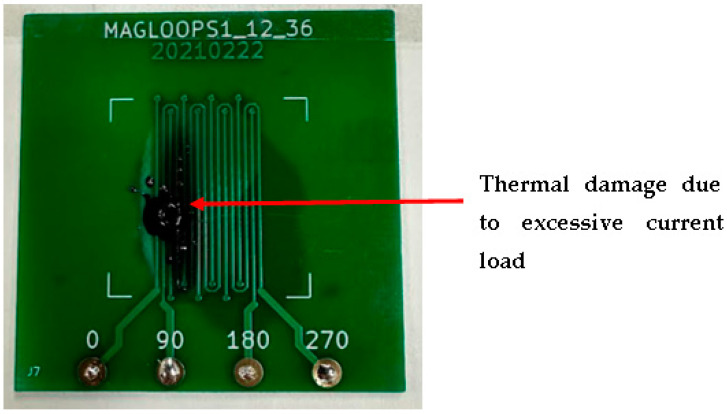
Photograph of the damaged chip during the forced convection setup.

As shown in Figure 9, at low current settings (0.25–1.5 A), the chip can survive the current loads without a heat sink involved. The need for a heat sink becomes evident at 2.5 A, where the chip experiences thermal breakdown (120 °C) at 2.5 A around 40 s. For our initial design, we constructed a forced convection setup consisting of a 3D-printed stand outfitted with a copper heat sink and central processing unit (*CPU*) fan. During experimental testing, it was concluded that the forced convection setup was not sufficient to continuously remove heat. In this setup, we were able to reach 3–4 A for a total time of 10 s before we exceeded 130 °C and the chip was damaged. Figure 10 below shows the result of a damaged chip after 10 s of heating with the forced convection setup.

**Figure 11:**
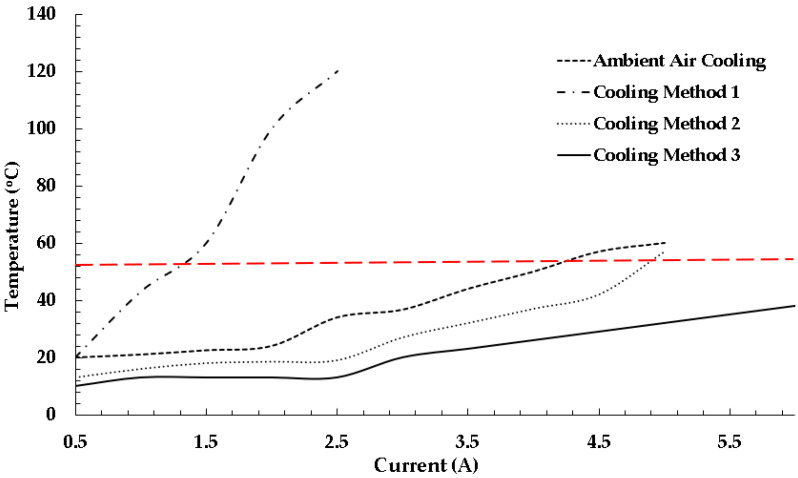
Averaged steady-state temperature for each current setting. Ambient air cooling is without a cooling device. Cooling method 1 is using a micro-cooler setup with water at 0 °C, cooling method 2 is using a Neslab bath circulator setup with the temperature set to 6 °C, and cooling method 3 is using a Neslab bath circulator setup with the temperature set to 0 °C.

At these current levels, heating of the programmable chip and microfluidic device and eventual thermal change to the electrodes are inevitable. As a result, we incorporated a cooling block and cooling water system into the experimental setup to maintain the electrodes as close to room temperature as possible during experimental runs. To test the effectiveness of the cooling water system, we performed a series of tests to determine the temperature response of the electrodes to the applied current, while the cooling water is active. A thermocouple is tightly connected directly atop a glass slide and placed on the electrodes to measure the temperature change for incremental currents. Figure 11 below shows the average temperature recording for each current setting for the four cooling methods (*ambient air cooling, the micro-cooler method with a setpoint of 0 °C (cooling method 1), the Neslab setup with a setpoint of 6 °C (cooling method 2), and the Neslab setup with a setpoint of −20 °C (cooling method 3))*). The temperature is recorded periodically for 3 min while currents of 2, 3, 4, 5, and 6 A are applied to the electrodes using an excitation frequency of 1 kHz. We observed that the temperature reaches a steady state value after around 30 s. More importantly, for currents less than 5 A, the cooling water keeps (*methods 1, 2, and 3*) the electrode chip temperature at room temperature to prevent the electrodes from burning or for the heat to affect the ferrofluid and sample being tested. 

As shown in Figure 11, cooling method 3 yielded the best temperature recording for a maximum current of 5.5 A. For the water-cooled experiments using the Neslab bath (*cooling method 3*), the bath was set to a temperature setpoint of −20 °C while water circulated through the cooling block and water bath during the experiment. The actual liquid temperature recorded at this setpoint temperature was 0 °C. Using this setup, we can maintain a sufficient temperature to avoid potential cell damage. The next section discusses the ferrofluid tailoring results.

### 3.2. Cobalt Ferrite Particle Synthesizing and Ferrofluid Tailoring Results

**Figure 12:**
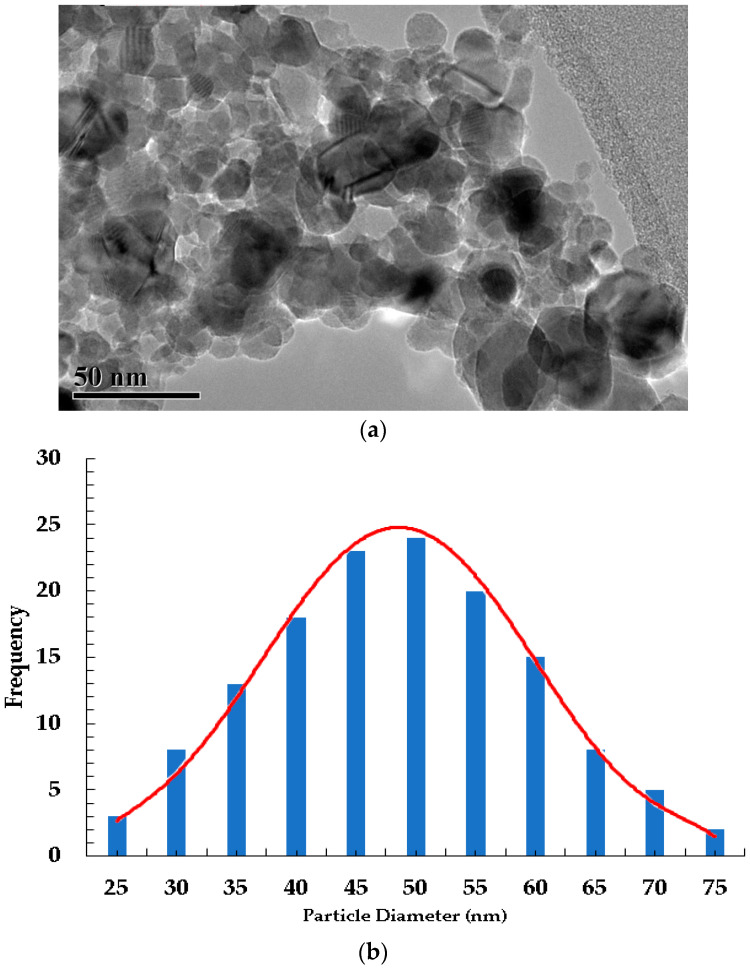
Individual particle sizes as extracted from TEM images for the first batch: (**a**) TEM image of the particles and (**b**) the log-normal distribution with a 48.53 nm average particle diameter.

In this work, we tailored a cobalt ferrite-based aqueous ferrofluid using a method described in detail in Section 3.3. Cobalt-ferrite possesses a high magnetic anisotropy energy density (*between 1.8 × 10^5^ and 3.0 × 10^5^ J/m^3^ for bulk material and up to 3.15 × 10^6^ J/m^3^ for nanoparticles* [37]). We conducted two experimental runs for tailoring cobalt ferrite particles. Figure 12 shows both the TEM images of the first batch of CoFe_2_O_4_ nanoparticles calcined at 600 °C for more than 10 h (*with an average crystallite size of about 48.53 nm as determined by XRD*) and the particle distribution histogram. 

**Figure 13:**
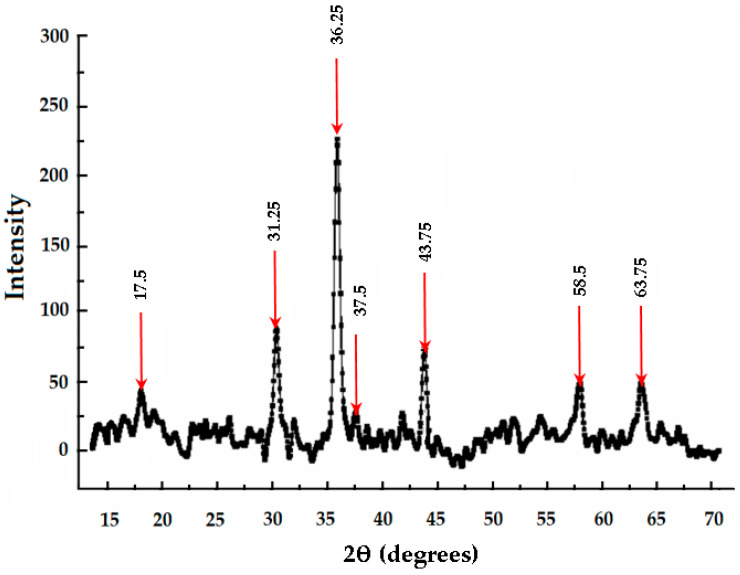
X-ray diffraction results for the 1st sample of cobalt ferrite particle tailoring.

We also performed X-ray diffraction studies on the synthesized cobalt ferrite microparticles. A plot of the X-ray diffraction results is shown in Figure 13. The X-ray diffraction pattern of the calcined powder synthesized shows that the final product is CoFe_2_O_4_ with the expected inverse spinel structure. No other phase/impurity was detected during the analysis. The size of the particles was determined by the Scherrer formula using the first two strongest peaks. The average sizes of the particles calcined at 600 °C were found to be approximately 50 ± 2 nm. 

The size distribution of these nanoparticles observed from the TEM images is shown in Figure 12b. The distribution appears to be symmetric (*Gaussian*) at about 48.53 nm, with a particle diameter range of 25–70 nm for this specimen, which is preferred. The maximum number lies between 45 and 55 nm, peaking at 48.53 nm, in good agreement with XRD crystallite size. Most of the particles appear spherical in shape; however, some elongated particles were also observed, as shown in the TEM image. Some moderately agglomerated particles as well as separated particles are present in the images.

**Figure 14:**
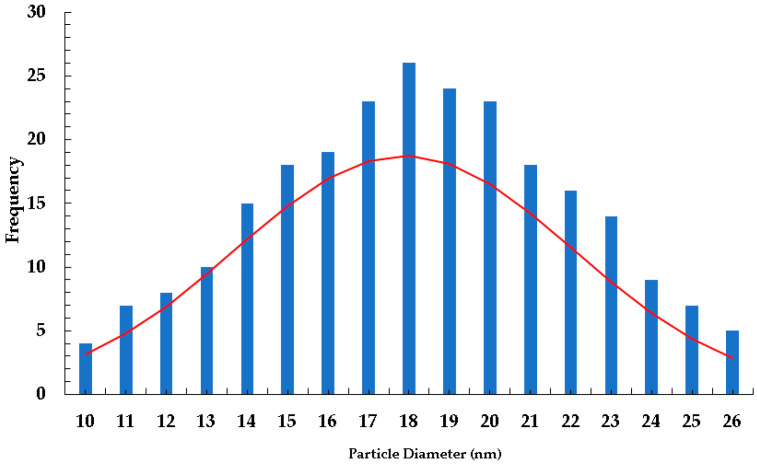
Histogram of particle sizes for the second batch with an average diameter of 17.9 nm.

We later observed for the second batch that increasing the NaOH rate and increasing the rpm above 3000 rpm led to smaller particle diameter sizes. In the second batch, we were able to achieve an average particle diameter of 17.9 nm. Figure 14 shows the histogram generated for the second batch. 

**Figure 15:**
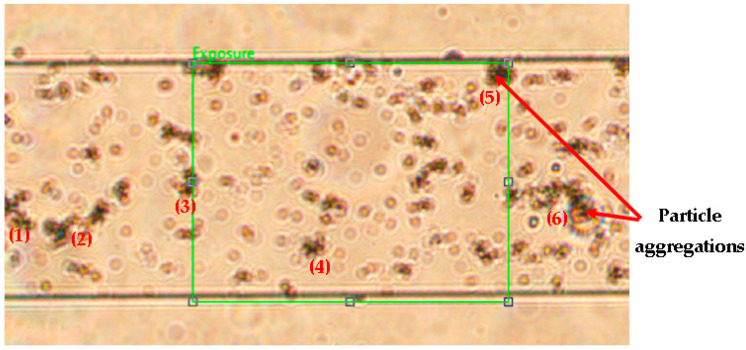
Microscope image of cobalt ferrite particles suspended in water in the PDMS single microchannel device. The numbered regions show the locations of the largest particle aggregations. The blurry particles represent particles closer to the bottom of the channel.

In this data set, the average diameter and standard deviation are 17.9 nm and 4.17 nm, respectively. This is ideal as compared to the first batch, as ferrofluids incorporating cobalt ferrite particles relax primarily by particle rotation (*Brownian motion*) for nanoparticles 5–20 nm in diameter. Prior to tailoring the cobalt ferrite and non-magnetic fluorescent microparticle ferrofluid, we suspended the tailored cobalt ferrite particles in deionized water, sonicated the mixture for 10 min, and injected a small sample of the mixture in a single microchannel device as shown in Figure 15. As shown in Figure 15, the cobalt ferrite particles are randomly suspended in the microchannel, with some particle aggregations observed (*numbered regions*). The blurry particles represent particles that are closer to the bottom of the channel. To minimize particle aggregations in the ferrofluid mixture, we sonicated succeeding ferrofluid mixtures for 20 min and noticed no further aggregations. 

**Figure 16:**
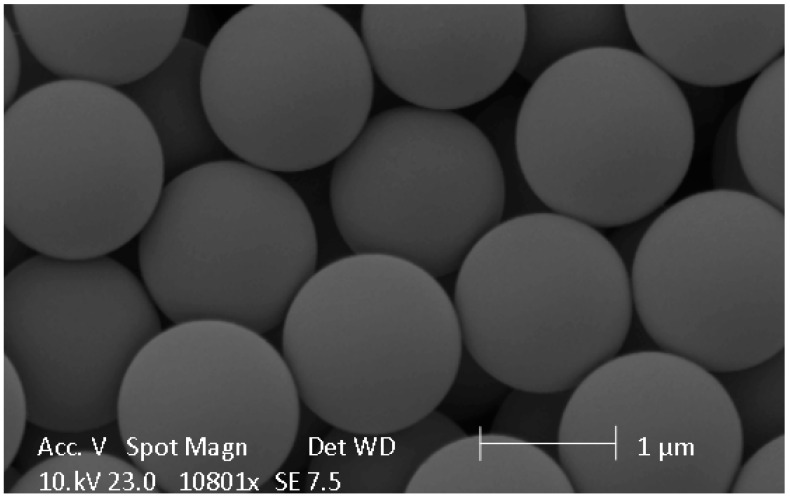
SEM images of 1 µm fluorescent microspheres.

For the non-magnetic microparticle manipulation experiments, we prepared a water-based ferrofluid comprised of cobalt ferrite microparticles, which were synthesized using the method described in Section 2.3. The viscosity of this ferrofluid was determined to be 1.75 cP at 20 °C. Our initial experiments involved characterizing the behavior of 1 µm diameter non-magnetic particles. Figure 16 below shows a scanning electron microscopy (*SEM*) image of the 1 µm diameter particles. The next section presents the particle dynamic results.

### 3.3. Particle Dynamic Characterization Studies

This section of the paper discusses the non-magnetic microparticle dynamic study results using image cross-correlation techniques. Prior works have incorporated simple algorithms for tracking particles using recursive particle tracking techniques. This work is unique in that we can use cross-correlation analysis to determine particle trajectories, particle/fluid velocities, streamlining of flow, and vorticity (*fluid/particle*) spin. To begin the analysis, an ensemble correlation of sparsely seeded steady-flow images is obtained from the videos taken during experimental runs and analyzed via cross-correlation. 

**Figure 17:**
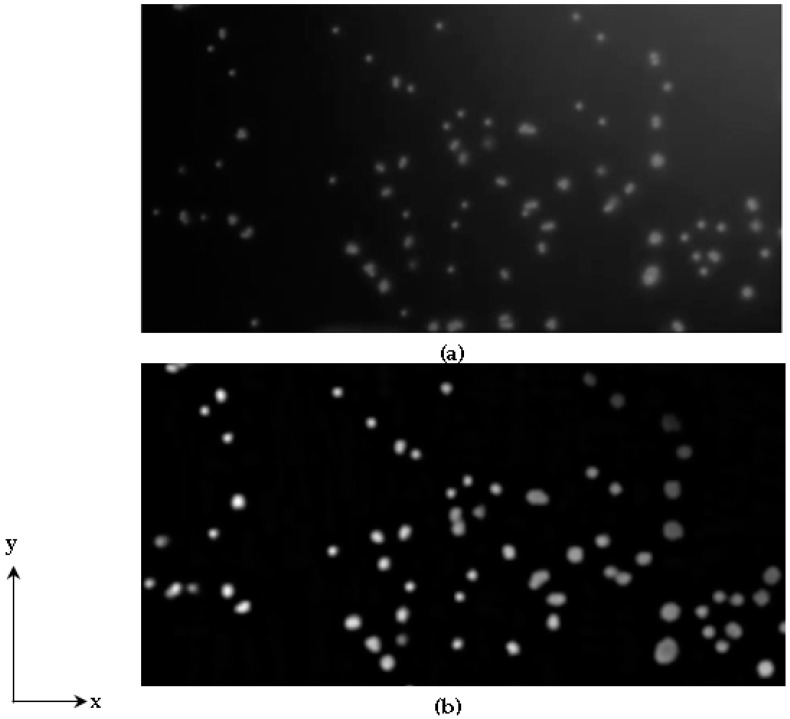
Particle image processing results in PIVlab for 4 µm diameter particles: (**a**) raw image and (**b**) PIVLAB pre-processed image with filters prior to cross-correlation analysis.

The resulting correlation matrices are averaged before a peak searching algorithm is used, resulting in a sufficient signal-to-noise ratio and high vector resolution for the low particle density images (*low concentration compared to PIV analyses*). We performed the ensemble correlation in PIVlab open-source software, which features all the advanced correlation techniques of the regular correlation. This ensemble correlation involved a multiple pass, window deformation, suppression of autocorrelation, and a background subtraction method for pre-processing particle/fluid flow images. To characterize the ferro-microfluidic device using fluorescent polystyrene microspheres, we conducted a series of experiments using ThermoFisher Scientific; fluorescent monodisperse with diameters ranging from 1 to 10 μm. Figure 17 below shows the result of processing a frame image using this method for 4 µm diameter particles. For this example, the 4 µm diameter particles are used as they are easy to visualize and track in sparsely seeded images. This study was conducted with an amperage of 4 A and a frequency of 10 Hz. 

**Figure 18:**
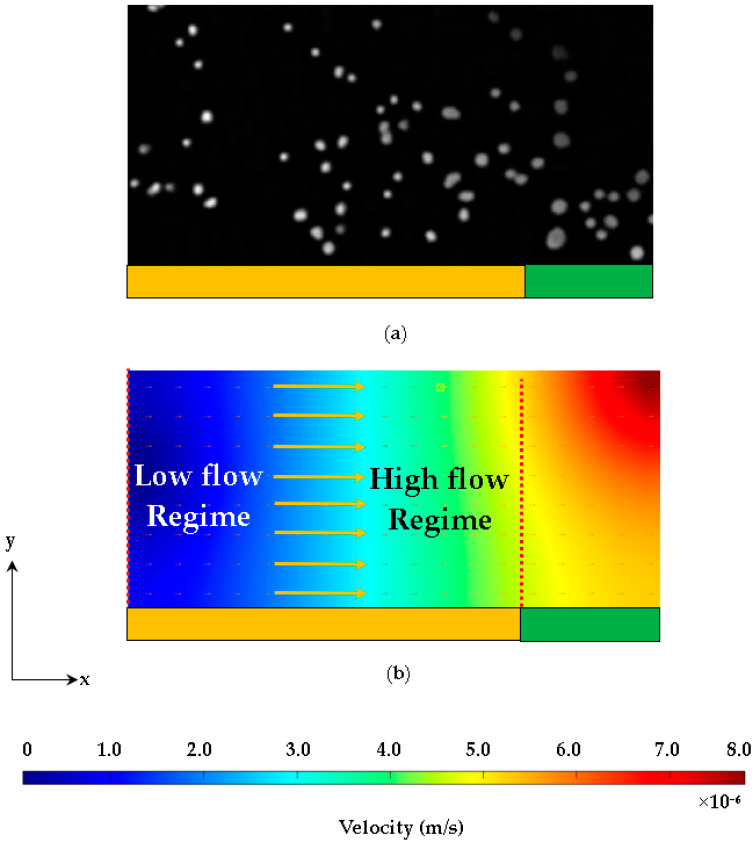
Overview of the particle image processing methodology in PIVlab. (**a**) Top view of 4 µm diameter particles moving in the micro-channel under excitation, and (**b**) The processed velocity contour image. The electrode (*gold*) and spacing (*green*) are shown below the figures.

For this study, the image post-processing consisted of calibrating the image with a reference line placed between the microchannel walls. The reference line was calibrated to the same length as the microchannel from wall to wall. This allowed the software to have a reference distance line for estimating velocity. After pre-processing the image, the images were resolved to velocity flow image contours. Figure 18 below shows an example of the processing methodology and the results using 4 µm diameter particles at 4 A and 10 Hz for the small spacing electrodes. As shown in Figure 18b, the velocity contour demonstrates that the low flow regime exists at the electrodes and increases at the electrode spacing gap. This is because the electrodes create the force necessary for the particles to be pushed to the top of the channel, gain momentum, and translate across the channel at a given frequency and amperage.

This phenomenon was observed in all studies, although some particles can be trapped at a given current, diameter, electrode size and spacing, and frequency. This is demonstrated later in Section 2.3.1. To characterize the ferro-microfluidic device, we needed to gain a baseline perspective of the influence of amperage amplitude, excitation frequency, electrode spacing, and particle size on particle dynamics. Section 3.3.1 and Section 3.3.2 provide a detailed discussion of the particle dynamics for the electrode spacing, amperage, and frequency settings.

#### 3.3.1. Large Electrode Spacing Study Results

For our initial studies, microspheres of a given individual size were initially mixed with the ferrofluid in small quantities and added to the microfluidic channel. We later introduced smaller 1 µm diameter particles with larger 10 µm diameter particles in the ferrofluid. For all studies, the particles were introduced into the microfluidic channel, and the microchannel inlet and outlet was closed off at both ends to prevent fluid motion. Microspheres near the roof of the microchannel were imaged and processed in PIVLab. A current was applied to the electrodes to generate a traveling wave magnetic field within the microfluidic channel due to the phase-differenced electrodes. Two electrode spacing studies are conducted in this *work (a small electrode spacing and a large electrode spacing)*. For both electrode spacing studies, the microspheres are randomly dispersed throughout the channel, prior to the current excitation. Depending on electrode spacing, the size-based particles can either be trapped between the electrodes or continuously pushed throughout the channel by the traveling wave magnetic field.

**Figure 19:**
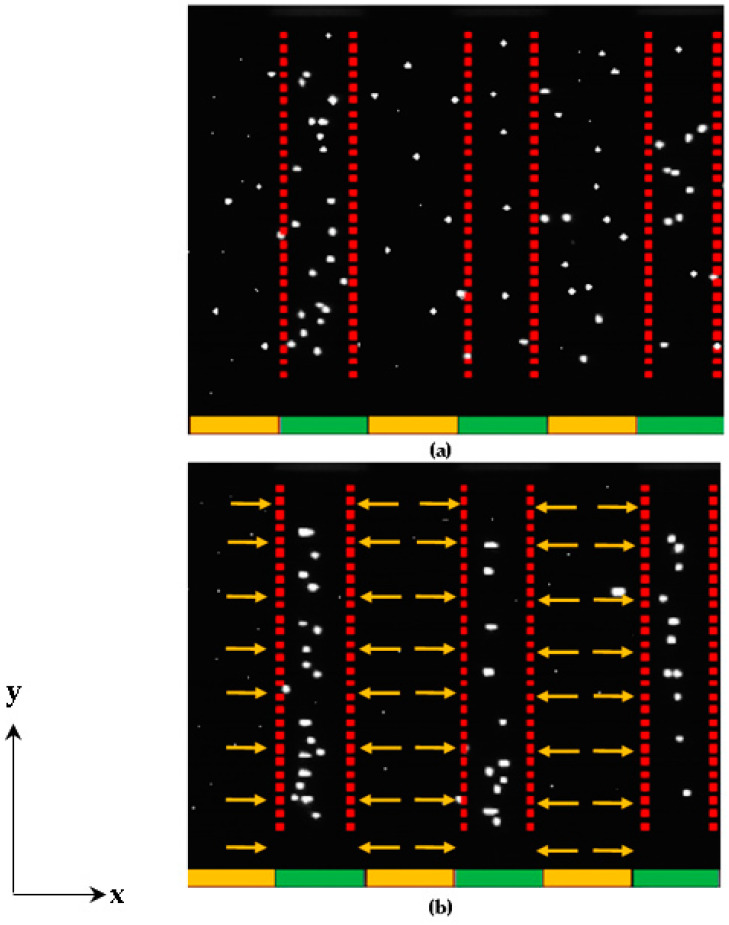
Top view of the microfluidic channel with the ferrofluid: (**a**) Image of 10 µm and 1 µm diameter microspheres randomly dispersed in the channel and (**b**) After the excitation, particles collect in the interelectrode spacing. The electrode (*gold*) and spacing (*green*) are shown below the figures.

For the large electrode spacing studies, we observed that when current is applied, the non-magnetic particles are pushed away from the electrodes to the top of the channel (*due to the magnetic force*), where they start to rotate (*due to the magnetic torque*). When the particles reach the top surface, their rotation leads to linear translation along the channel. It was observed that at low frequencies, the microspheres would localize between the excitation electrodes, where the repulsive forces due to magnetic field gradients form a local minima. Figure 19 below illustrates this phenomenon.

**Figure 20:**
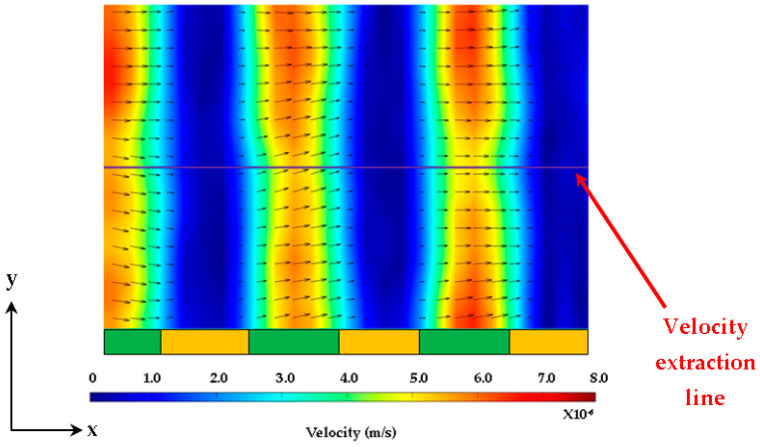
Velocity contour of 10 particles moving between the electrodes at 10 Hz and with a large electrode spacing. The electrode (*gold*) and spacing (*green*) are shown below the figure.

As shown in Figure 19, the particles are randomly distributed in the channel. When the field is turned on at 6 A and with a frequency of 10 Hz, the larger particles tend to localize in the space between the electrodes. The smaller 1 µm diameter particles appear to not be affected. Figure 20 shows a cross-correlation processed image from PIVlab of the 10 µm diameter particles moving to the electrode spacing under the influence of the electrode excitation and frequency. As shown in Figure 20, the flow magnitudes appear to be the greatest at the electrodes. As mentioned previously, this is because the electrodes create the force necessary for the particles to be pushed to the top of the channel, gain momentum, and translate across the channel at a given frequency and amperage. Figure 20 shows a plot of the extracted velocity plotted in the middle of the channel and across the electrode region. The extraction line is shown in Figure 20 (*red arrow pointing to the line*).

**Figure 21:**
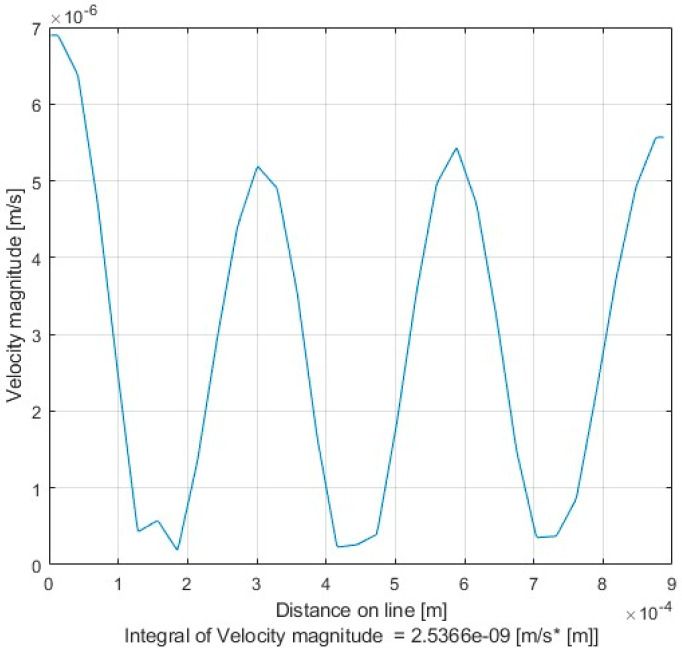
Plot of the velocity distribution in the middle of the channel and across the electrode region.

**Figure 22:**
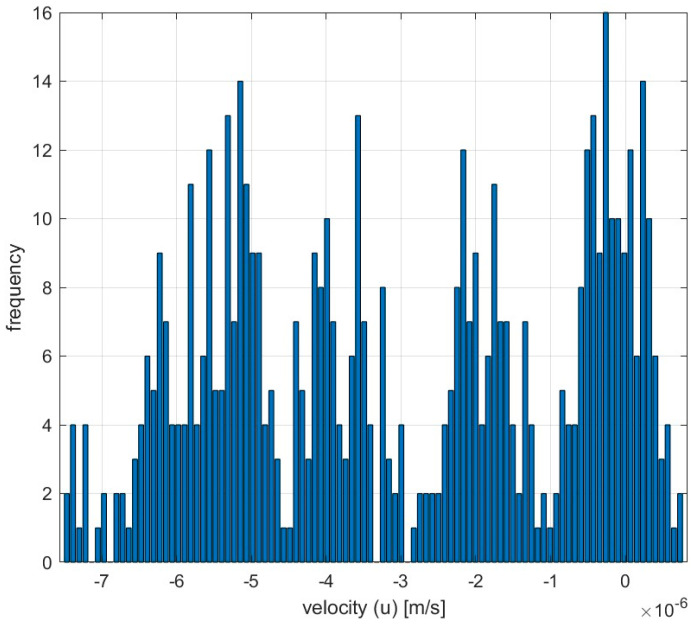
Histogram of the velocity distribution from the image dataset.

As shown in Figure 21, the velocity profile has a sinusoidal profile similar to the trend of the phase-differenced input currents. The plot indicated that the velocity increases in the electrode spacing regions and decreases at the electrodes. Figure 22 shows a histogram of the velocity amplitudes in the processed image.

Unlike the velocity contours, the histogram provides information on the frequency distribution of velocity magnitudes for the entire data set of images. As shown in Figure 22, the highest velocity observed has the least occurrence, whereas the lowest velocity of no movement occurs the most. This indicates that the particles move from the highest force region at a high magnitude velocity and then come to rest between the electrodes.

#### 3.3.2. Small Electrode Spacing Study Results

**Figure 23:**
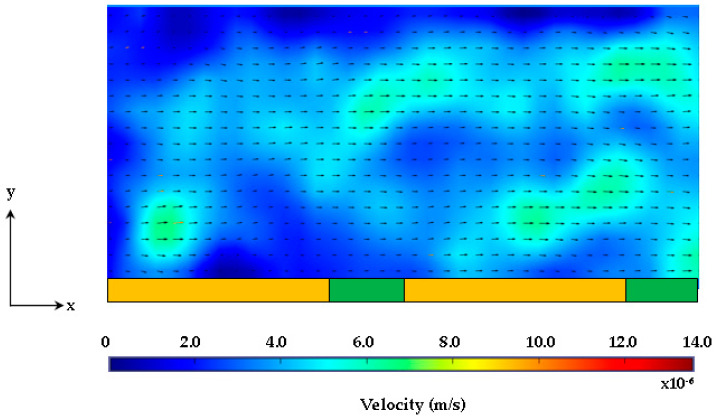
Velocity contour of 1 µm diameter particles under 750 mA and 10 Hz.

For the small electrode spacing experimental studies, we observed that when current is applied to the electrodes, the particles tend to move to the top surface of the channel and translate along the roof of the channel. Unlike the large electrode spacing chip experimental studies, the smaller electrode spacing leads to more kinetic energy in the fundamental mode of the traveling wave excitation. As mentioned previously, it also reduces the lateral field gradient between the electrodes. In this manner, smaller electrode spacings will lead to faster microsphere travel and a reduction in the critical frequency for trapping. Figure 23 below illustrates this effect. 

**Figure 24:**
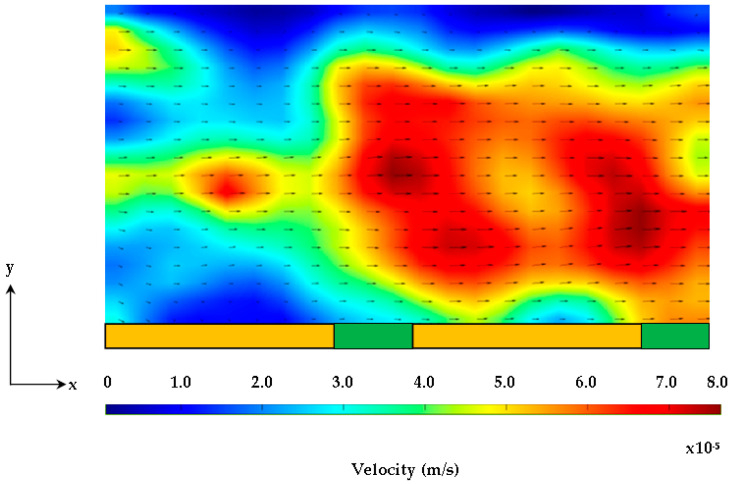
Velocity contour of 10 µm diameter fluorescent particles at 4 A and 10 Hz.

Figure 23 shows a processed velocity contour of 1 µm diameter fluorescent particles moving in the microchannel at a low amperage setting of 750 mA and a frequency setting of 10 Hz. Surprisingly, at a low amperage setting, the 1 µm diameter particles can be continuously pushed through the channel (*from left to right*). It appears that the small spaced electrodes create a ratcheting effect of continuously pushing particles downstream regardless of the current setting for the 1 µm diameter particles. Figure 18 shows the velocity contour of the 1 µm diameter fluorescent particles moving in the microchannel at a low amperage setting of 4 A and a frequency setting of 10 Hz. The velocity magnitude has increased due to the increased amperage. It also appears that the electrodes create the ratcheting effect for larger particles as well. As shown in Figure 24, the velocity magnitude is lower at the first electrode and then increases at the electrode space and increases at the second electrode. 

**Figure 25:**
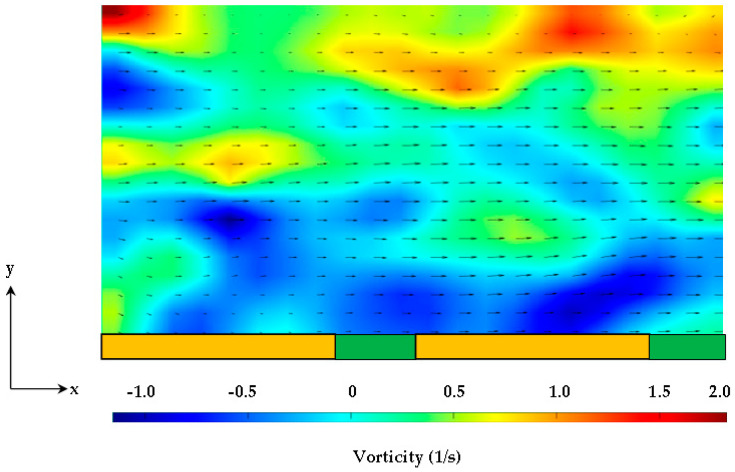
Vorticity contours of 10 µm diameter fluorescent particles at 4 A and 10 Hz.

**Figure 26:**
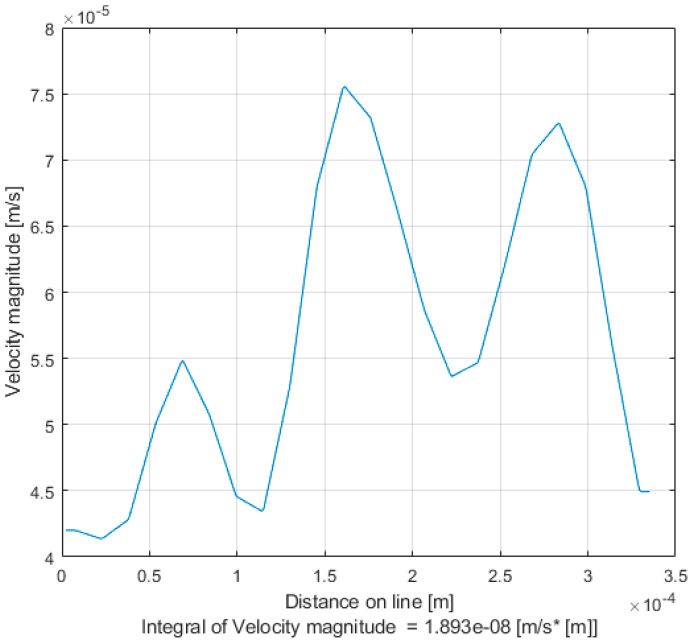
Velocity distribution extraction from the centerline of the 10 µm diameter particle study at 4 A and 10 Hz.

**Figure 27:**
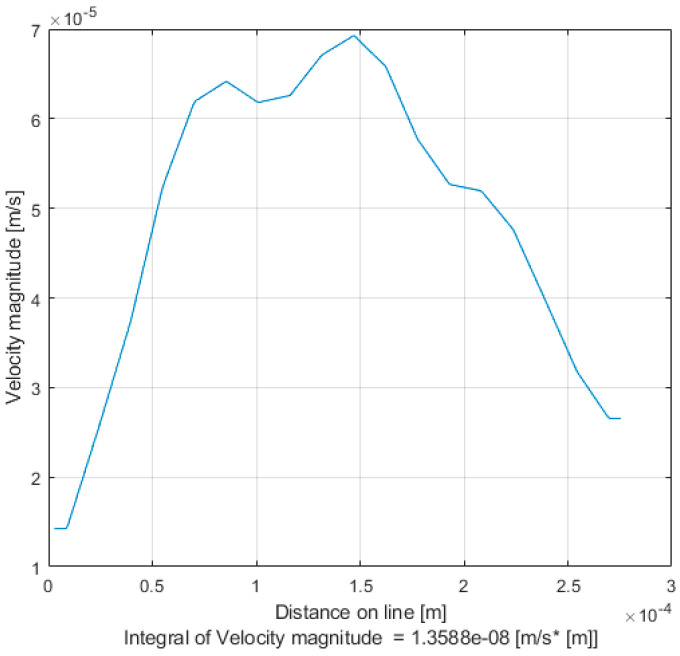
Velocity profile extraction from the middle of the first electrode for the 10 µm diameter particle study at 4 A and 10 Hz.

Figure 25 shows the vorticity contour for the same image of 10 µm moving in the microchannel. Vorticity is a measure of the spin of the fluid [38,39,40]. As shown in the contour, the highest spin is experienced near the edges of the electrodes, which indicates the regions where the magnetic push and pull forces are the highest. This push-and-pull effect creates the ratcheting effect mentioned previously. This is also demonstrated in the centerline velocity distribution plotted in Figure 26. The velocity centerline plot shows that the highest velocity occurs near the edges of the second electrode. The velocity profile (*wall to wall*) is extracted at the middle of the first electrode and shown in Figure 27. The velocity profile shows that the flow is laminar and slightly fully developed. The no-slip condition is not captured in the image due to the glare of scattered light.

**Figure 28:**
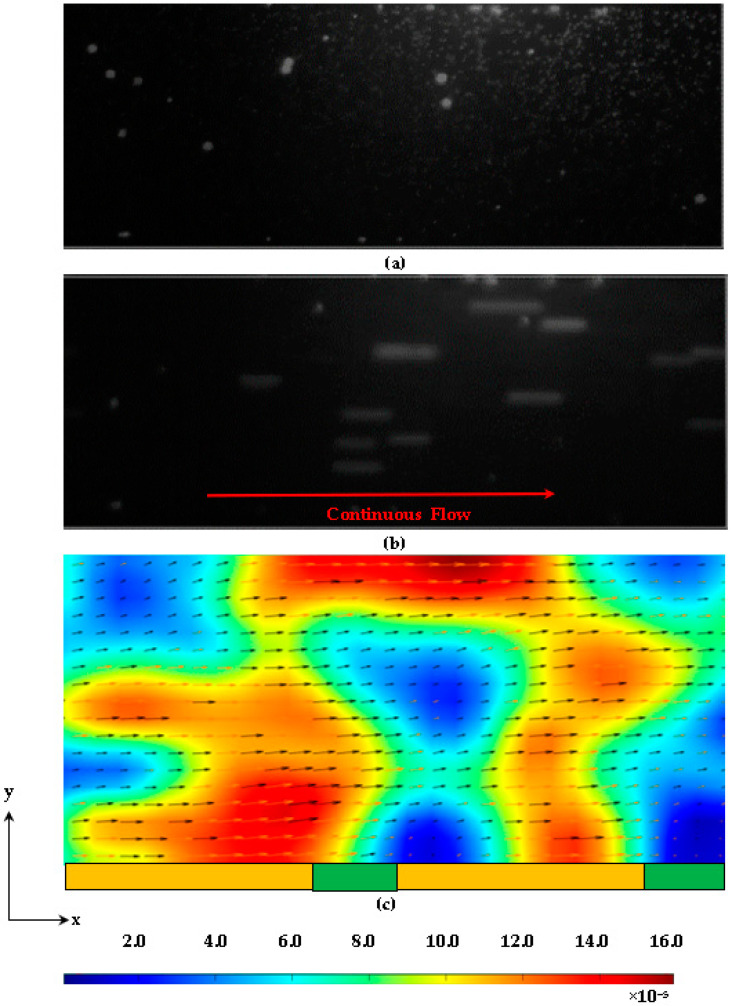
Particle images and contours obtained at 6 A and 10 Hz before and during excitation: (**a**) image of particles randomly dispersed before the field is turned on, (**b**) particle images obtained during excitation, and (**c**) the processed velocity contour of 1 um diameter fluorescent particles at 6 A and 10 Hz.

Figure 28 shows the images of 1 µm and 10 µm diameter particles randomly dispersed in the ferrofluid within the microchannel. The current is set to 6 A with a frequency of 10 Hz. Similar to the 1 µm diameter particle studies, the particles continuously translate along the roof of the channel. This behavior was consistent at various frequencies of 10, 100, 1000, and 10,000 Hz.

## 4. Conclusions

The design and development of a simple traveling wave ferro-microfluidic device and system rig purposed for the potential manipulation and magnetophoretic separation of cells in a water-based ferrofluid were presented. This work consisted of designing a heat dissipation system for effectively removing heat during particle manipulation studies, fabricating cobalt ferrite nanoparticles, tailoring a water-base ferrofluid for particle manipulation studies, and conducting particle dynamic studies for characterizing the behavior of particles under an applied amperage, frequency, and underlying electrode spacing. 

Experimental studies were carried out using two different diameter sizes of non-magnetic (*fluorescent microsphere*) particles. These studies have demonstrated the ability to trap large, 10 µm diameter particles at low frequencies using large electrode spacing and continuously pushing particles through the channel with smaller electrode spacing, regardless of the particle size. The results reported in this work demonstrate a proof of concept for magnetophoretic manipulation and separation of magnetic and non-magnetic nanoparticles in a simple ferro-microfluidic device. This work is a design and proof-of-concept study. From a heat transfer perspective, the design reported in this work is an improvement over existing traveling wave ferro-microfluidic designs in that heat is efficiently removed from the circuit board to allow a range of input currents and frequencies to manipulate non-magnetic particles. The results reported in this work establish that the developed ferro-microfluidic device may potentially be used as an effective platform for microparticle and cellular manipulation and sorting. 

The major finding in this work is that the velocity of the microspheres depends on the excitation frequency, current amplitude, electrode spacing, and their position with respect to the underlying electrodes. The key contributions of this work are:

1. A method for tailoring preferred size range (10–20 nm) magnetic cobalt ferrite magnetic nanoparticles.
2. The development of a ferro-microfluidic device for potentially separating cells and magnetic microparticles without detrimental thermal effects.
3. The development of a water-based ferrofluid with magnetic and non-magnetic particles as surrogates for biological cells.
4. The design and development of a rig for producing the electric field within the ferro-micro fluidic device for magnetizing the magnetic nanoparticles and manipulating nanoparticles in static and dynamic flow while efficiently removing heat from the electrode base.
5. A method for measuring the surrounding fluid and particle velocity, vorticity, and characterizing particle dynamics in the developed ferrofluid.
6. An approach for separating magnetic and non-magnetic nanoparticles that could potentially be an advantage is that particle manipulation will not rely on labeling or surface modification, significantly reducing operation time and cost compared to conventional approaches.

Future work will include:

1. Using a particle tracking code for particle dynamics studies as opposed to a cross-correlation methodology.
2. Refining the chip base design to incorporate thicker copper electrodes (*in the direction of the thickness of the PCB*) for smaller resistance in the electrodes. This would possibly result in less heat for any given excitation current.
3. Investigate different metallic electrode core materials to provide a more effective path for heat to flow away from the magnetic excitation pattern.
4. Develop different electrode patterns (*square and curved*) with varying sizes on one chip to characterize the behavior of particles under varying amperage and frequency conditions for both static and dynamic flow. Conducting further studies using this approach will allow us to establish the feasibility of this approach for potentially separating cells.
5. Conduct particle dynamic characterization studies using commercial ferrofluids such as EMG 700 with varying electrode patterns, amperage settings, frequency settings, and non-magnetic particle sizes for both static and dynamic flow.
6. Tailor biocompatible ferrofluids and conduct cellular-based studies such as separating bacteria from magnetic particles in dynamic flow studies.

## Data Availability

The data from this work will be shared and made available upon request to the corresponding author.
